# A Facile Approach for Fabricating Microstructured Surface Based on Etched Template by Inkjet Printing Technology

**DOI:** 10.3390/polym10111209

**Published:** 2018-10-31

**Authors:** Jiazhen Sun, Chenghu Yun, Bo Cui, Pingping Li, Guangping Liu, Xin Wang, Fuqiang Chu

**Affiliations:** Key Laboratory of Pulp, Paper, Printing & Packaging of China National Light Industry, Key Laboratory of Printing & Packaging Materials and Technology of Shandong Province, School of Light Industry and Engineering, Qilu University of Technology (Shandong Academy of Sciences), Jinan 250353, China; chenghuyun@qlu.edu.cn (C.Y.); cuibo@qlu.edu.cn (B.C.); lpp@qlu.edu.cn (P.L.); lgp@qlu.edu.cn (G.L.); wangxin@qlu.edu.cn (X.W.); fqchu@126.com (F.C.)

**Keywords:** inkjet printing, etched template, microstructured surface, anisotropic wettability

## Abstract

Microstructures are playing an important role in manufacturing functional devices, due to their unique properties, such as wettability or flexibility. Recently, various microstructured surfaces have been fabricated to realize functional applications. To achieve the applications, photolithography or printing technology is utilized to produce the microstructures. However, these methods require preparing templates or masks, which are usually complex and expensive. Herein, a facile approach for fabricating microstructured surfaces was studied based on etched template by inkjet printing technology. Precured polydimethylsiloxane substrate was etched by inkjet printing water-soluble polyacrylic acid solution. Then, the polydimethylsiloxane substrate was cured and rinsed, which could be directly used as template for fabricating microstructured surfaces. Surfaces with raised dots, lines, and squares, were facilely obtained using the etched templates by inkjet printing technology. Furthermore, controllable anisotropic wettability was exhibited on the raised line microstructured surface. This work provides a flexible and scalable way to fabricate various microstructured surfaces. It would bring about excellent performance, which could find numerous applications in optoelectronic devices, biological chips, microreactors, wearable products, and related fields.

## 1. Introduction

Microstructures are playing an important role in manufacturing functional devices, due to their unique properties, such as wettability or flexibility [1,2]. Recently, various microstructured surfaces have been fabricated to realize functional applications in manipulating droplets, wearable devices, and energy conversion [3,4,5]. The microstructured surfaces are commonly prepared by some methods for constructing various functional materials on the surfaces, which could be used to realize the applications [6,7]. Previously, there has been much effort to fabricate microstructured surfaces for some special functionality, including laser etching, photomask template processing, microcontact printing, and so on [8,9,10,11]. Maeng et al. created a 3D microcavity array via a reactive ion etching (RIE) process, which contributed to the increment of the area capacitance [5]. Lee et al. used a double photolithography method to obtain the mogul-patterned substrate, which provided more possibilities for device flexibility [12]. Liu et al. obtained a separate metallic network structure by depositing metal material on a template by using laser-direct writing patterning techniques [13]. However, the abovementioned methods require elaborately fabricating templates or expensive vacuum deposited masks that restrict practical applications. Therefore, it is highly desirable to conceive a simple and effective route to fabricate substrates with various microstructures.

As a solution processing method, printing technology is widely used to fabricate various functional patterns [14]. Especially, inkjet printing technology has been proved to be a versatile patterning technique because of its direct-writing and mask-free properties. With the high resolution, cost saving, and efficient use of materials [15,16,17,18,19], inkjet printing technology has aroused wide attention as a simple and efficient way for patterning functional materials. Various functional materials are deposited by inkjet printing to fabricate transistor circuits, light-emitting polymer displays, solar cells, sensor arrays, stretchable heater, optical devices, and other devices [20,21,22,23,24,25,26,27]. Meanwhile, viscoelastic substrate and sacrificial polymer etching could produce various micro/nanostructures for special applications. Szilasi et al. conducted an in-depth study of the selective etching of polydimethylsiloxane (PDMS) using potassium hydroxide and sodium hydroxide [28]. Liu et al. used soft lithography to obtain PDMS with a rectangular channel as the top layer of the device [29]. Wang et al. deposited polymethyl methacrylate (PMMA) onto a silica substrate as a sacrificial mask to deposit a metal film [30]. Melzer et al. used polyacrylic acid (PAA) as a sacrificial layer to transfer the magnetic sensor directly to the elastomer [31]. Agarwal et al. performed large-scale nanoimprinting based on the different solubility of the sacrificial PAA layer between monovalent and divalent cations [32]. In this study, a facile approach for fabricating microstructured surfaces was studied based on etched template by inkjet printing technology. Precured polydimethylsiloxane substrate was etched by inkjet printing water-soluble polyacrylic acid solution. Then, the polydimethylsiloxane (PDMS) substrate was cured and rinsed, which could be directly used as a template for fabricating microstructured surfaces. Surfaces with raised dots, lines, and squares, were facilely obtained using the etched templates by inkjet printing technology. Furthermore, controllable anisotropic wettability was exhibited on the raised line microstructured surface. This work provides a flexible and scalable way to fabricate various microstructured surfaces. It would bring about excellent performance, which could find numerous applications in optoelectronic devices, biological chips, microreactors, wearable products, and related fields.

## 2. Materials and Methods

### 2.1. Materials

PDMS (precursor and curing agent): (Sylgard 184, Dow Corning, Midland, MI, USA).

Polyethylene glycol terephthalate (PET): (Beijing Daxiang Plastic Co., Ltd., Beijing, China).

Ethanol: (Beijing Chemical Co., Ltd., Beijing, China).

Polyacrylic acid (PAA, Mw = 1800, Sigma-Aldrich, Saint Louis, MO, USA).

Polyvinyl alcohol (PVA, 1788, Sigma-Aldrich, Saint Louis, MO, USA).

Deionized water (generated by Milli-Q water purification system, Beijing, China).

### 2.2. Preparation of Water-Soluble Polymer Ink

Polyacrylic acid (PAA, MW = 1800, Sigma-Aldrich) dissolved in water/ethanol mixed solvent (with volume ratio 20:80) was used as the sacrificial ink. The concentration of the ink was kept as 16 wt %.

### 2.3. Preparation of Inkjet Printing Substrate

PDMS base was mixed with a curing agent in the proportion of 15:1, by weight, to make PDMS precursor (Sylgard 184). The precursor was put into a centrifuge to remove air bubbles (1000 rpm, 2 min), then it was put onto a PET-supporting layer by spin-coating at 800 rpm for 20 s. The height of the liquid PDMS film was about 150 μm. As the too-thin film would be affected by the PET supporting layer, and the too-thick film would induce an unstable thermoresponsibility, the thickness of PDMS film was chosen as 150 μm in this experiment. Subsequently, the PDMS precursor with reducing agent was precured in a 70 °C oven for a given time, and used as the inkjet printing liquid substrate [33,34].

### 2.4. Inkjet Printing Water-Soluble Polymer Ink

The inkjet printing process was performed via Dimatix Materials Printer (FUJIFILM DMP-2800 series, Tokyo, Japan) with a 25 μm nozzle and 10 μm inkjet droplet precise distance controlled with Dimatix Drop Manager Software. The diameter of inkjet droplet was about 25 μm, with the 25 μm nozzle. In this work, the factor influencing the diameter of inkjet droplet was just considered with the pushing stage, that the diameter of droplet was confined by the diameter of nozzle. The environmental temperature of inkjet printing was 25 °C. The humidity of the environment was 65%. A single nozzle was used during the inkjet printing process. Inkjet printing frequency was 5.0 kHz. A customized waveform was utilized in inkjet printing with piezoelectric maximum 30 V, and pulse width 8.5 μs.

### 2.5. Fabrication of Inkjet Printed Template

Then, the inkjet-printed substrates were heated at 70 °C for another 1 h to completely solidify the PDMS precursor. After rinsing with water, water-soluble polymer could be removed from the surface of substrates. The inkjet-printed substrates could be used as templates. When replicating materials were spin-coated on the inkjet-printed substrates and, subsequently, uncovered slowly, microstructured patterns of these materials could be immediately constructed. The replicating materials should satisfy the condition that the materials could transfer onto a film with evaporating or curing, such as PDMS or PVA. Therefore, the microstructured surfaces were facilely fabricated.

### 2.6. Characterization

Structures of inkjet-printed patterns were investigated by SEM (JSM-7500, Tokyo, Japan), with a scanning voltage of 5 kV. Optical micrographs were acquired by optical microscope (Olympus MX40, Tokyo, Japan). Contact angles of the patterned substrates were measured using a contact angle system (Dataphysics OCA20, Filderstadt, Germany) at ambient temperature. The height profiles of the patterned surface were determined in a surface profiler (Kosaka ET4000, Tokyo, Japan).

## 3. Results and Discussions

### 3.1. Fabricating Microstructured Surfaces Based on Etched Template by Inkjet Printing Technology

**Figure 1:**
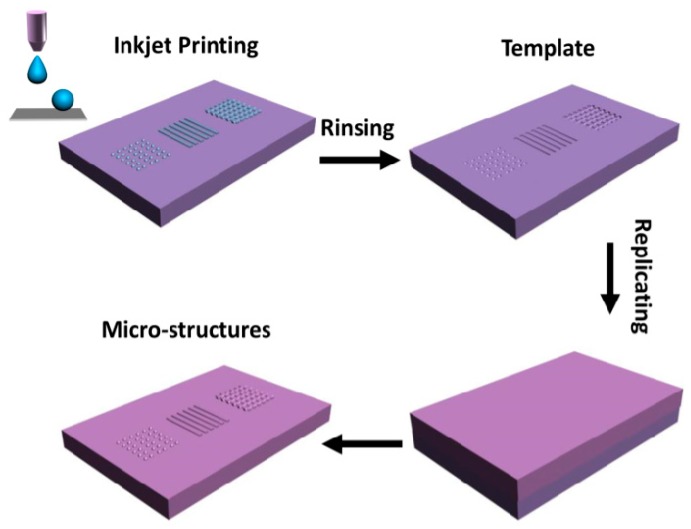
Scheme of fabricating microstructured surface based on etched templates by inkjet printing technology.

Figure 1 demonstrates the template fabricating and microstructure patterning processes. Firstly, PDMS substrates are etched into templates by inkjet printing technology. The fabricating process is that PDMS precursor is spin-coated onto a supporting layer, and precured into viscoelastic state. Water polyacrylic acid (PAA) ink is inkjet-printed onto the viscoelastic surface to form semi-wrapped structures. The inkjet droplet could imprint the viscoelastic precured PDMS substrate and generate a semi-wrapped deposit. When the precured PDMS liquid film is connected to the evaporating PAA droplet, the depositing resolution of inkjet droplets would be improved with the viscoelastic pressure of substrate. The existing position of deposited structure is influenced by the fluidity and viscoelasticity. If the precured PDMS liquid film has a high fluidity, the water-soluble deposits would exist in the PDMS substrate. If the precured PDMS liquid film has a high viscoelasticity, the water-soluble deposits would exist on the PDMS substrate. Accordingly, water-soluble deposits with semi-wrapped structure are generated on the PDMS surface with a proper cure temperature and time. After solidification of the substrate and rinsing the deposited PAA, the etched substrate is produced as the template. When various materials are spin-coated on the template, the etched template would be replicated onto the surface of various materials. Then, the materials are uncovered slowly from the templates, and the surfaces with functional microstructures would be facilely fabricated. Therefore, patterned microstructures of these materials could be immediately constructed based on etched templates by inkjet printing technology.

**Figure 2:**
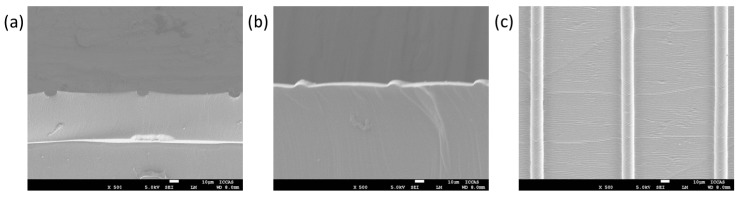
Morphologies of the etched template and microstructured surface, based on the etched template. (**a**) Cross-section morphology of the etched template. (**b**) Cross-section morphology of the replicated microstructure based on the etched template. (**c**) Top view morphology of the replicated microstructure based on the etched template.

**Figure 3:**
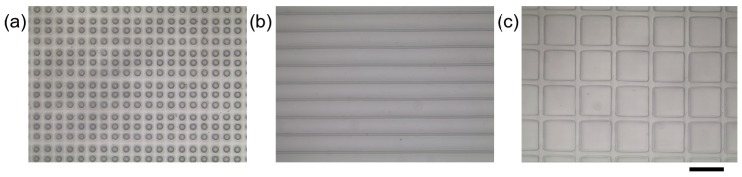
Morphologies of fabricated microstructured surfaces based on the etched template. (**a**) The microstructured surface with raised dot. (**b**) The microstructured surface with raised line. (**c**) The microstructured surface with raised square. Scale bar: 200 μm.

Figure 2 shows the morphologies of etched template with line pattern and the fabricated microstructured surface with raised line, which were characterized by SEM. The etched resolution is 10 μm, and the resolution of replicated microstructure is also 10 μm. In this process, the PDMS was also used as the material of microstructured surface, because PDMS is commonly applied as various functional surfaces with its transparency, flexibility, and good biocompatibility. Meanwhile, the morphologies of microstructured surfaces with raised dots, lines, and squares, which were fabricated based on the etched templates (Figures S1 and S2) by inkjet printing technology, were characterized by SEM (Figure 3). The raised microstructures were replicated from multilayer etched templates. The diameters were improved with the multilayer etched templates. The smooth edge of the template, after etching, could be presented with the high magnification images of raised dots (Figure S3). The results show that the microstructured surfaces with raised dots, lines, and squares, could be facilely achieved with a large scale and high resolution, using the etched templates by inkjet printing technology. Structuring of complicated designs could be fabricated by combining the different inkjet printing templates. One gradient microstructure with two types of raised line were fabricated based on two types of etched line template (Figure S4). The method of producing template by inkjet printing technology provides an efficient way to produce microstructured surfaces.

### 3.2. Controllable Fabrication of Microstructured Surfaces Based on Etched Template by Inkjet Printing Technology

**Figure 4:**
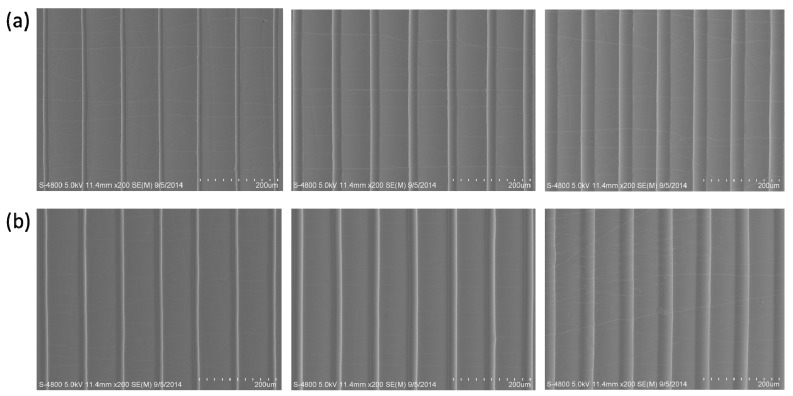
Morphologies of etched line templates and the microstructured surface with raised line based on multilayer inkjet-printed microtemplates. (**a**) The etched templates with different inkjet printing layers (1, 3, 5 layers from left to right). (**b**) The replicated microstructures with different inkjet printing layers (1, 3, 5 layers from left to right).

**Figure 5:**
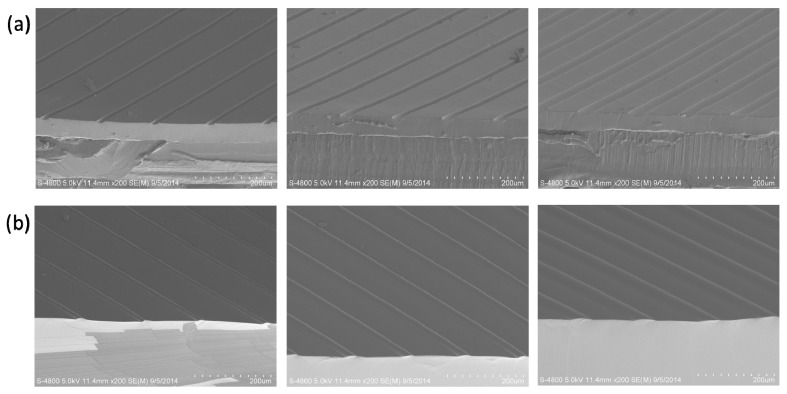
Cross-section morphologies of etched line templates and the microstructured surface with raised line based on the etched templates. (**a**) The etched templates with different inkjet printing layers (1, 3, 5 layers from left to right). (**b**) The replicated microstructures with different inkjet printing layers (1, 3, 5 layers from left to right).

**Figure 6:**
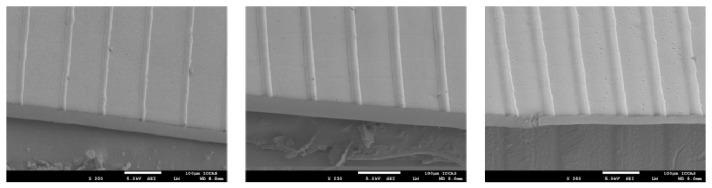
Morphologies of fabricated line microstructured surfaces of polyvinyl alcohol (PVA) based on etched line templates with different inkjet printing layers (1, 3, 5 layers from left to right).

The morphologies of the etched templates are determined by the interactions between the inkjet-printed droplets and the viscoelastic PDMS substrates, which could be adjusted by inkjet printing layers. As shown in Figure 4, the templates with different printing layers were fabricated, and the microstructures with different morphologies were also fabricated based on the changed templates. The diameter of etched template could be adjusted with the inkjet printing layers, which could be used to obtain differently replicated microstructured surfaces. To show the groove structure clearly, the cross-morphologies of the templates and microstructures were shown in Figure 5. Profiles of the raised line structures have been characterized by profilometer (Figure S5). Meanwhile, the microstructured surfaces with different materials were also realized. Figure 6 shows that the microstructured surfaces of polyvinyl alcohol (PVA) could also be fabricated based on the changing templates.

### 3.3. Anisotropic Wetting Behavior on Fabricated Microstructured Surfaces

**Figure 7:**
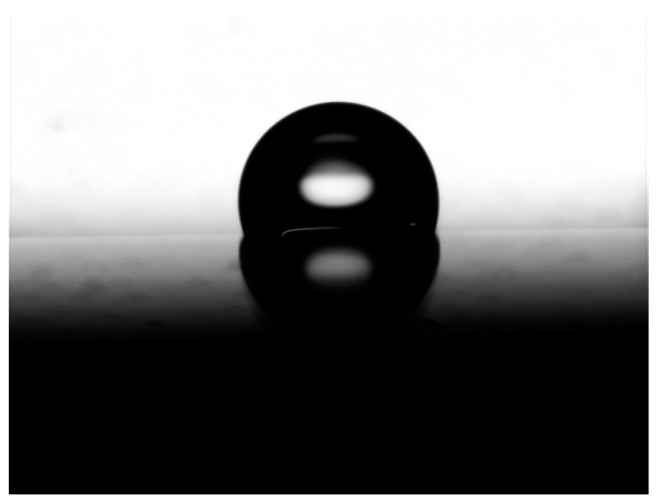
Wetting behavior of a 5 μL water droplet on a flat PDMS surface with static wetting contact angle about 107.2°.

**Figure 8:**
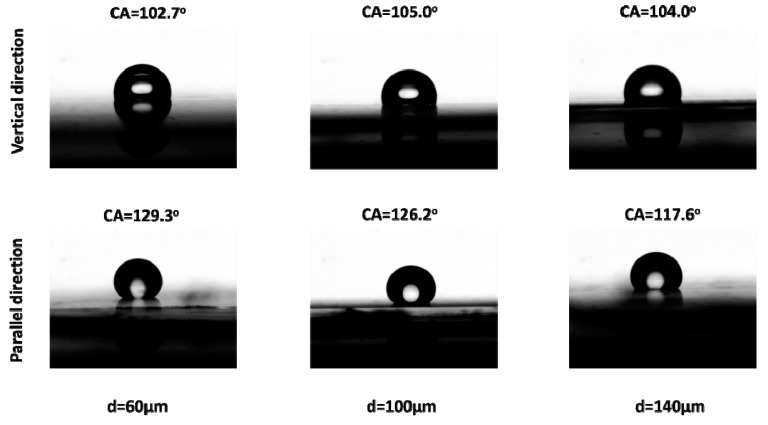
Wetting behavior of droplet on the microstructured surface with raised line in parallel direction and vertical direction with 60, 100, and 140 μm line spacing.

Furthermore, the anisotropic wetting behavior of droplet was exhibited on the microstructured surface with raised lines. The microstructured surface with raised lines could be facilely obtained based on the above method, which would present the advantage of the approach for fabricating microstructured surface based on etched template by inkjet printing technology. Then, the wetting behaviors of water droplets were characterized on the fabricated microstructured surfaces. As shown in Figure 7, the static wetting contact angle of a 5 μL water droplet was about 107.2° on the unprocessed PDMS surface. When the water droplet was residing on the fabricated microstructured surface with raised line, the static wetting contact angles with different directions were investigated. The wetting behavior on the microstructured surface with raised line was studied with 60, 100, and 140 μm line spacing, respectively. It was found that the wetting behavior of droplet on the microstructured surface appeared as a large difference in the parallel direction and vertical direction in Figure 8. Meanwhile, the wetting behavior of droplet on the microstructured surface also appeared as a large difference with different line spacing. When the micrometer scale PDMS is treated with the nanometer scale structure, the hierarchical structure would exhibit some special wetting behavior of superamphiphobic or sliding wettability. Therefore, a flexible and speedy way to fabricate anisotropic surface was realized based on the etched template by inkjet printing technology.

## 4. Conclusions

In this study, microstructured surfaces were fabricated based on etched template by inkjet printing technology. Water-soluble polyacrylic acid solution was used as inkjet printing ink, and then precured polydimethylsiloxane substrate was etched by the inkjet printing technology. After the polydimethylsiloxane (PDMS) substrate was cured and rinsed, the etched surface could directly serve as template. Then, the fabricating process of functional microstructured surface was studied based on the etched template by inkjet printing technology. Surfaces with raised dots, lines, and squares were facilely obtained using the etched templates by inkjet printing technology. Furthermore, the microstructured surface with raised lines could be facilely obtained based on the above method, which would present the advantage of the approach for fabricating microstructured surface based on etched template by inkjet printing technology. The controllable anisotropic wetting behavior of droplets was exhibited on the processing surface with the microstructure of the raised line. A simple method was developed for rapid fabrication of functional surfaces. It is a flexible and speedy way to fabricate functional microstructured surface based on the etched template by inkjet printing technology. These microstructured surfaces would present excellent performances, which could find numerous applications in optoelectronic devices, lab-on-chip devices, microreactors, and related fields.

## Supplementary Materials

The following are available online at http://www.mdpi.com/2073-4360/10/11/1209/s1, Figure S1: SEM image of etched dot template for fabricating microstructured surfaces with raised dots, Figure S2: SEM image of etched square template for fabricating microstructured surfaces with raised squares, Figure S3: Optical image of the high magnification images of raised dot microstructured surface, fabricated based on the etched template, Figure S4: Structuring of complicated designs with one gradient microstructure fabricated based on two types of the etched line template, Figure S5: Profiles of the raised line microstructured surface fabricated based on the etched template.
