# Supplementary material for: A Facile Approach for Fabricating Microstructured Surface Based on Etched Template by Inkjet Printing Technology
Source: Polymers (Basel). 2018 Oct 31;10(11):1209. doi: 10.3390/polym10111209 (PMC6290637; doi:10.3390/polym10111209)
Supplement: Supplementary file 1 [file polymers-10-01209-s001.pdf]

Supporting information

A Facile Approach for Fabricating Microstructured Surface Based on Etched Template by Inkjet Printing Technology

Jiazhen Sun *, Chenghu Yun, Bo Cui, Pingping Li, Guangping Liu, Xin Wang and Fuqiang Chu

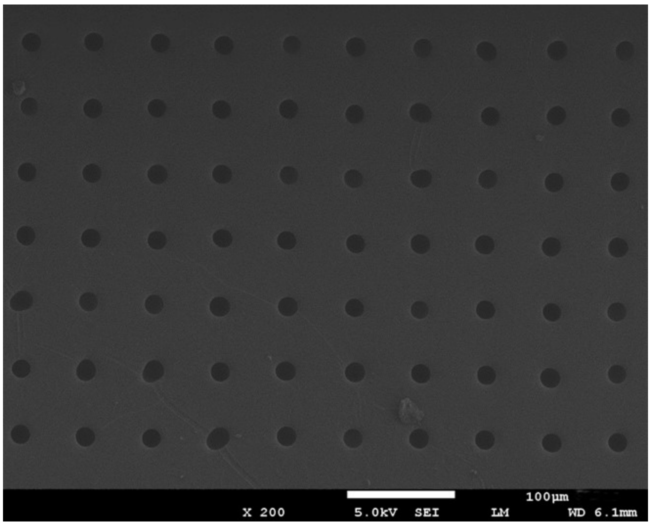

Figure S1. SEM image of etched dot template for fabricating microstructured surface with raised dot.

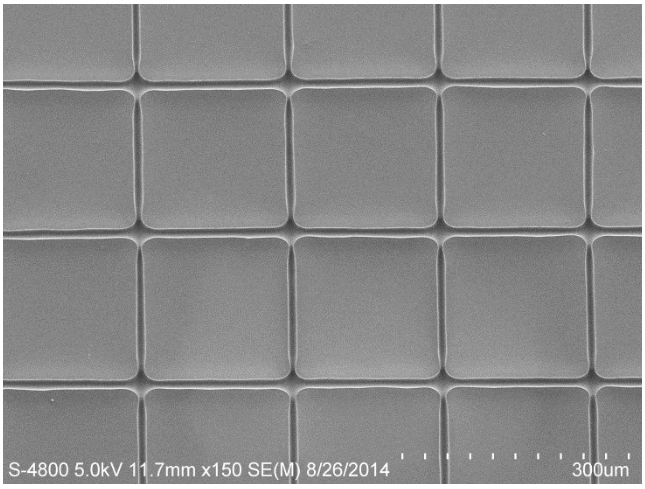

Figure S2. SEM image of etched square template for fabricating microstructured surface with raised square.

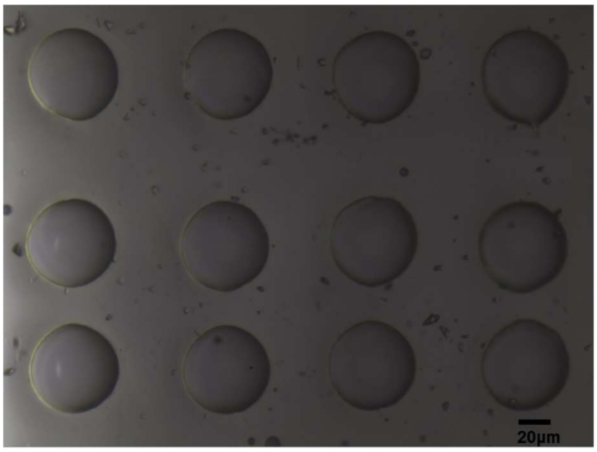

Figure S3. Optical image of the high magnification images of raised dot microstructured surface fabricated based on the etched template.

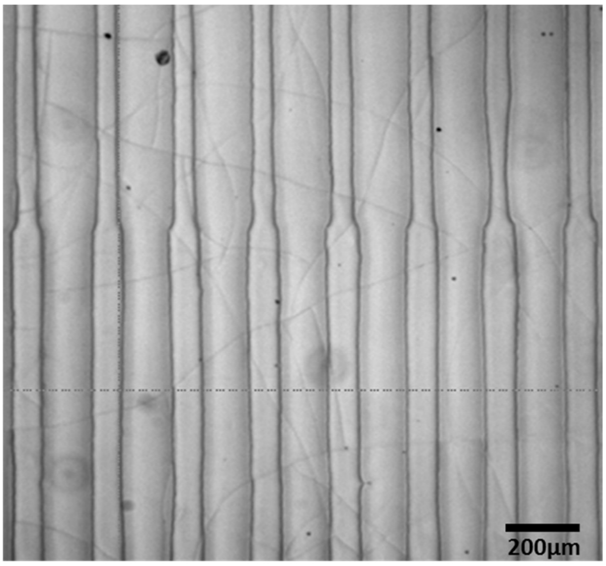

Figure S4. Structuring of complicated designs with one gradient microstructure fabricated based on two types of the etched line template.

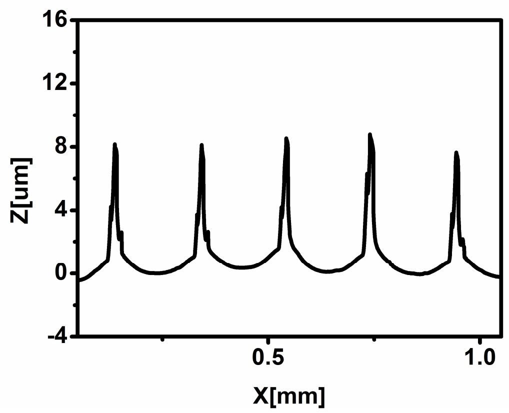

Figure S5. Profiles of the raised line microstructured surface fabricated based on the etched template.
